# A Beginner's Guide to Arterial Spin Labeling (ASL) Image Processing

**DOI:** 10.3389/fradi.2022.929533

**Published:** 2022-06-14

**Authors:** Patricia Clement, Jan Petr, Mathijs B. J. Dijsselhof, Beatriz Padrela, Maurice Pasternak, Sudipto Dolui, Lina Jarutyte, Nandor Pinter, Luis Hernandez-Garcia, Andrew Jahn, Joost P. A. Kuijer, Frederik Barkhof, Henk J. M. M. Mutsaerts, Vera C. Keil

**Affiliations:** ^1^Ghent Institute for Functional and Metabolic Imaging (GIfMI), Ghent University, Ghent, Belgium; ^2^Institute of Radiopharmaceutical Cancer Research, Helmholtz-Zentrum Dresden-Rossendorf, Dresden, Germany; ^3^Department of Radiology and Nuclear Medicine, Amsterdam UMC, location VUmc, Amsterdam, Netherlands; ^4^Amsterdam Neuroscience, Brain Imaging, Amsterdam, Netherlands; ^5^Hurvitz Brain Sciences Research Program, Sunnybrook Research Institute, Toronto, OT, Canada; ^6^Department of Radiology, University of Pennsylvania, Philadelphia, PA, United States; ^7^School of Psychological Science, University of Bristol, England, United Kingdom; ^8^Dent Neurologic Institute, Buffalo, Amherst, NY, United States; ^9^Department of Neurosurgery, University at Buffalo, Buffalo, NY, United States; ^10^fMRI Laboratory, Department of Biomedical Engineering, University of Michigan, Ann Arbor, MI, United States; ^11^Queen Square Institute of Neurology and Center for Medical Image Computing, University College London, London, United Kingdom; ^12^Cancer Center Amsterdam, Imaging and Biomarkers, Amsterdam, Netherlands

**Keywords:** arterial spin labeling, MRI, image processing, cerebral blood flow, graphical user interface, perfusion, processing pipeline

## Abstract

Arterial spin labeling (ASL) is a non-invasive and cost-effective MRI technique for brain perfusion measurements. While it has developed into a robust technique for scientific and clinical use, its image processing can still be daunting. The 2019 Ann Arbor ISMRM ASL working group established that education is one of the main areas that can accelerate the use of ASL in research and clinical practice. Specifically, the post-acquisition processing of ASL images and their preparation for region-of-interest or voxel-wise statistical analyses is a topic that has not yet received much educational attention. This educational review is aimed at those with an interest in ASL image processing and analysis. We provide summaries of all typical ASL processing steps on both single-subject and group levels. The readers are assumed to have a basic understanding of cerebral perfusion (patho) physiology; a basic level of programming or image analysis is not required. Starting with an introduction of the physiology and MRI technique behind ASL, and how they interact with the image processing, we present an overview of processing pipelines and explain the specific ASL processing steps. Example video and image illustrations of ASL studies of different cases, as well as model calculations, help the reader develop an understanding of which processing steps to check for their own analyses. Some of the educational content can be extrapolated to the processing of other MRI data. We anticipate that this educational review will help accelerate the application of ASL MRI for clinical brain research.

## Introduction

Arterial spin labeling (ASL) has established itself as a magnetic resonance imaging (MRI) technique for measuring cerebral perfusion (1). While gadolinium-contrast-based dynamic-susceptibility contrast (DSC) MRI remains the most frequently clinically used method to measure brain perfusion (2). ASL has undeniable advantages: it does not require an exogenous tracer such as gadolinium, and the labeled water molecules are not confined to the intravascular space. ASL is therefore immediately repeatable, avoids pharmacological side effects (3), and has economic advantages compared to DSC, dynamic contrast-enhanced MRI perfusion (DCE) or even positron-transmitting tomography (PET). These more invasive techniques follow acquisition techniques fundamentally different from ASL, which in return demand a different, and not easily comparable image processing strategy.

Significant progress has been made to improve ASL acquisition techniques, and to improve data quality and robustness (1, 4, 5). Advanced ASL techniques, such as multi-post-labeling delay (multi-PLD) ASL, have been developed to correct for physiological differences between patients in blood arrival time (6).

Expertise in ASL acquisition is slowly increasing within the clinical setting. However, MRI vendor-provided software usually is a rigid “black box” to deliver single-subject data, and therefore is not adequate for the needs of a group-based study (7, 8). An in-house processing pipeline, on the other hand, requires substantial programming skills and introduces variability among centers (8, 9). Programmers do not necessarily have experience with the special requirements for ASL processing, which demand consideration of physiology and the clinical condition under investigation, while clinicians often lack the expertise in image processing.

Understanding the relevance of image processing steps in the context of physiology and pathology is a prerequisite to conducting ASL-based studies. A graphical user interface (GUI) can facilitate a user's interaction with the processing pipeline — as it visually outlines any available options and can provide suggestions on the fly — but does not help understand the rationale behind the ASL processing steps (10–12).

This educational review aims to empower those who want to gain an understanding of ASL image processing for scientific use coming from both a clinical and an image processing background. The most important processing steps of ASL quantification will be explained, along with their relevance for improving data quality and avoiding artifacts and false results.

## ASL Acquisition in A Nutshell

There are excellent reviews on ASL acquisition techniques and potential clinical applications (13, 14). In addition, there is an extensive dictionary of ASL terminology developed by the Open Science Initiative for Perfusion Imaging (OSIPI) Task Force 4.1 (15), and some basic technique-related abbreviations are defined in this online supplement (Supplementary Material 1; ASL abbreviation directory).

Here we, therefore, provide only a concise overview of how ASL data is acquired. In short, cerebral blood flow (CBF) is determined by several cardiovascular parameters, such as the heart rate and blood pressure, as well as the blood volume and its composition, like the hematocrit, the vessel anatomy, and finally the oxygen metabolism itself (so-called neurovascular coupling) (16, 17). Typical CBF ranges from 50 to 70 m/100 g brain tissue/minute — abbreviated as mL/100g/min — in cortical gray matter (GM) and about 20 mL/100g/min in white matter (WM) in young and healthy adults (4, 18).

**Figure 1:**
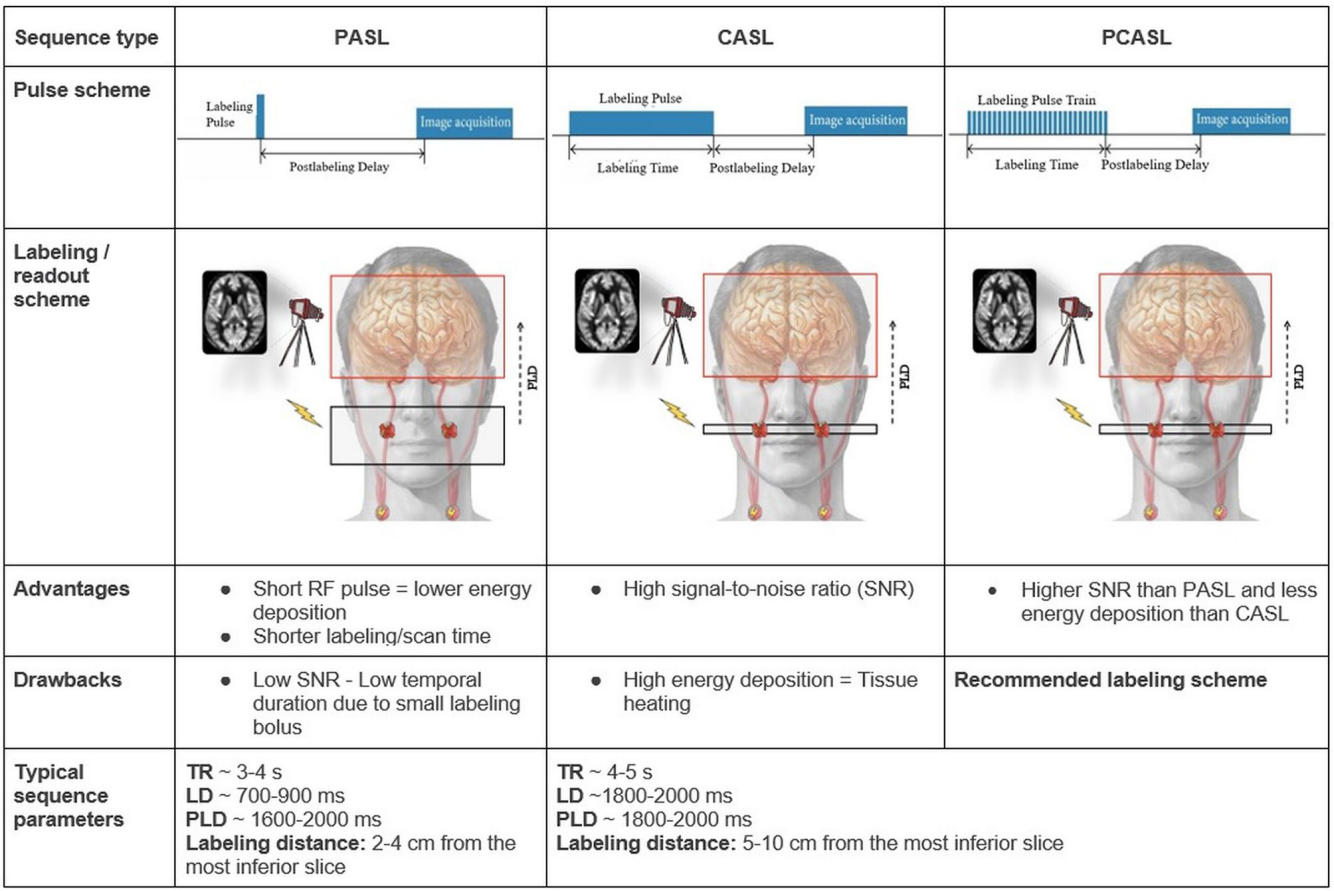
Overview of the PASL, CASL, and PCASL techniques The red H circles in the schematic overview images represent water protons in the blood that get labeled — depicted as red X marks — by a radiofrequency pulse (flash) in the labeling plane in the internal carotid arteries. Other extracranial arteries are labeled too but are not depicted here. Advantages and drawbacks are in comparison between the techniques. LD: labeling duration; RF: radiofrequency; TR: repetition time.

The major clinically applied labeling techniques are the spatial-selective pulsed (PASL) and pseudo-continuous (PCASL) labeling strategies (4). We will therefore not elaborate on other labeling strategies. Each ASL sequence consists of two parts: the control and the label image acquisition. The control image is acquired without prior labeling. For the label image, a so-called labeling pulse is first applied on the level of the cervical arteries, the labeling plane (Figure 1).

“Labeling” means changing the blood magnetization (more specifically the spin magnetization is inverted but it suffices to know that the magnetization is changed with respect to the rest of the brain). “Spins” are jargon for protons (H^+^), which are twice present in water (H_2_O), the main component of blood, and hence used as ASL MRI “contrast”. For all ASL techniques, the acquisition begins with labeling inflowing blood. Importantly, the labeled blood starts to relax immediately with a blood T1 time of about 1,650 ms for normal hematocrit levels at three Tesla (4). After this labeling, a “waiting” time, called post-labeling delay (PLD), is introduced to allow the labeled blood to flow into the brain vessels and capillaries. At this point, the label image is acquired. Control and label images are acquired using the exact same readout and form a pair. Their signal subtraction provides a unitless perfusion-weighted image (19). Additionally, a perfusion calibration image, known as the M0 image, is usually acquired to know the basic tissue magnetization. So the M0 image aims to translate how high a signal (measured in arbitrary units, a.u.) is for a certain amount of protons. Tissue magnetization can be used to estimate the baseline (or 0) reference magnetization (hence called M0), whose images are used to quantify CBF in mL/100g/min.

Typically, the relative difference between the control and label images is only 1-2% of the raw M0 magnetization resulting in a poor signal-to-noise ratio (SNR). To improve the quality of the final CBF images, many repetitions of the control and label images are acquired. Another important option in the ASL acquisition is called “background suppression”. Background suppression applies additional radiofrequency pulses between the blood labeling and image readout that reduce the background tissue signal while minimally affecting the perfusion component. Thereby, the detrimental effect of head motion on a CBF image is reduced (20).

Many pathology-induced perfusion alterations are typically too small to be visually assessed in individuals and only become statistically significant on a group level, doing a cohort or population analysis, e.g., comparing CBF in Alzheimer's patients and healthy volunteers.

ASL can be performed for single or multiple PLDs. Multi-PLD ASL provides more information about the temporal dynamics of the label arrival in the tissue and can improve the accuracy of the CBF measurement. Single PLD sequences are typically used in clinical practice, often due to time constraints, wider availability, and simplicity of both measurement and evaluation.

**Figure 2:**
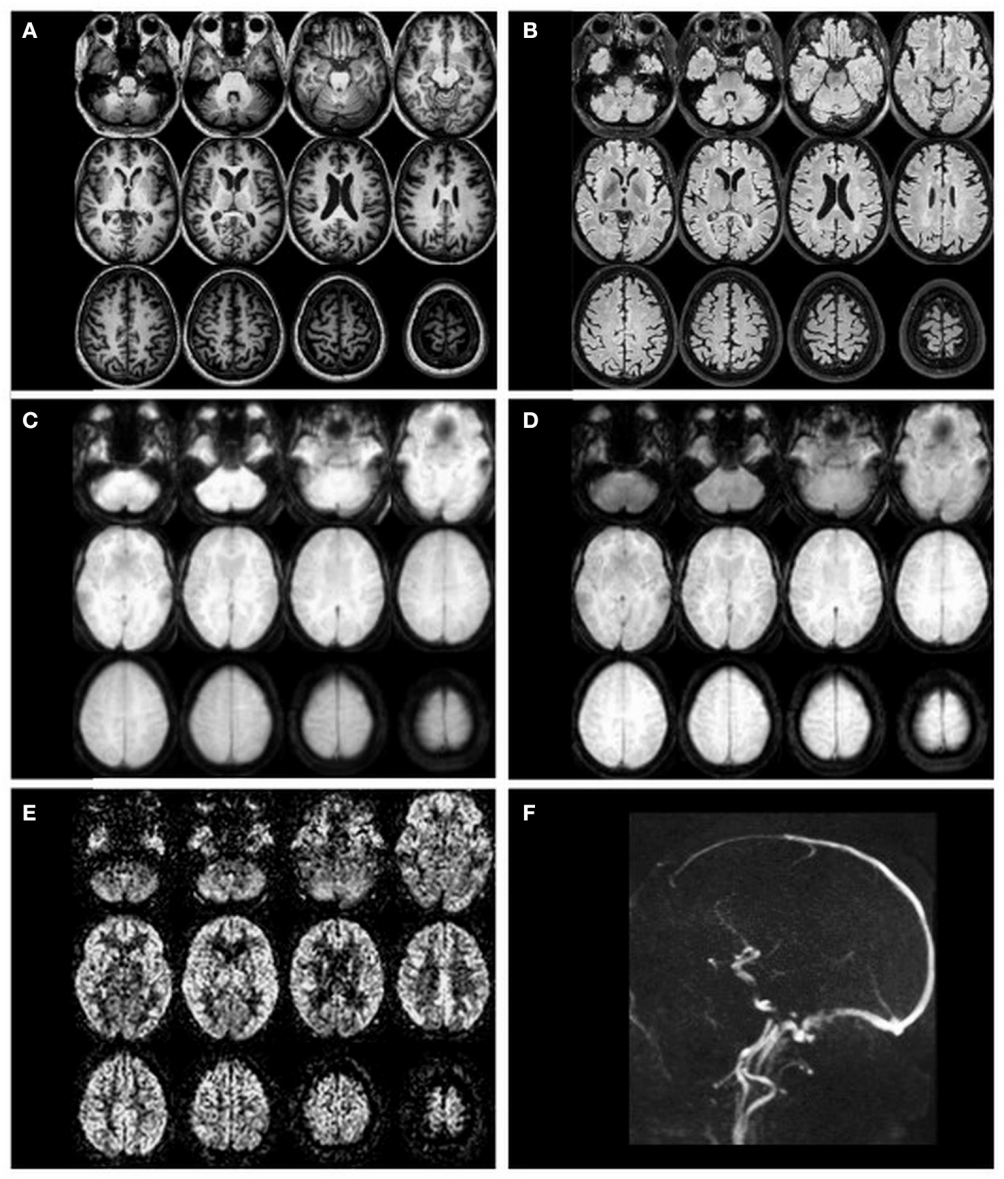
Example transverse slices of intermediate and final ASL images as quality control provided by ExploreASL, from a single healthy volunteer (female, 79 years of age) with 2D EPI PCASL sequence. **(A)** T1-weighted structural image in standard space; **(B)** T2 FLAIR image (pre-processed); **(C)** M0 image; **(D)** an average raw non-subtracted ASL control image; **(E)** the final CBF image; **(F)** a sagittal 2D time-of-flight vessel scout used to position the labeling plane.

Besides the ASL images, a structural image is needed to assess CBF in anatomical regions of interest (ROI). A 3D T1-weighted sequence with strong gray-to-white matter contrast and 1 mm isotropic voxel spatial resolution is commonly used. The acquisition of T2-weighted (or T2-weighted fluid-attenuated inversion recovery, T2 FLAIR) images is optional and can be used to improve processing. Visual examples of the mentioned images are provided in Figure 2.

**Learning points:**

1. ASL is a gadolinium-free MRI perfusion technique
2. Perfusion differences often can only be detected on a group level
3. T1-weighted structural images are usually needed for group-level analyses

## Processing Technology

There are many ASL processing technologies, so-called pipelines, and toolboxes, available online, some of which provide a GUI. This is an interface of a program that allows easy user interactions through visual elements like buttons, sliders, or check-boxes. This interface enables a user without any programming skills to control a software program but also hides the complexities of the program actions in the background. A non-exhaustive overview of GUIs for ASL processing, including technical details is provided in Table 1. For a curated list of ASL processing pipelines, the OSIPI Taskforce 1.1 inventory is recommended (25). This OSIPI inventory provides an overview of supported MRI vendors, accepted input data formats, and requirements for computer operating systems as well as additional software for both pipelines with and without GUI. While ASL pipelines might differ in quality and analysis options, most are able to process basic single PLD PCASL and PASL data, including M0 images and structural T1-weighted images.

**Table 1:** GUIs for ASL processing.

**Name**	**Free for academic use**	**Dicom conversion tool integrated**	**ASL-BIDS support**	**Supported ASL types**	**Segmentation basis**	**PVC used**
ASL-MRICloud (10, 21)	yes	no	NIfTI supported, JSON not used	PASL, PCASL, multi-PLD	T1w-MRICloud tool	no
ASLtbx (22)	yes	no	NIfTI supported	PASL, PCASL, multi-PLD	SPM-based	yes
BASIL asl_gui (23)	yes	no	NIfTI supported, ASL-BIDS in development	PASL, PCASL, multi-PLD, time-encoded, velocity-selective	FSL-FAST	yes
ExploreASL GUI (12)	yes	yes, using dcm2niix	yes, full support of ASL-BIDS	PASL, PCASL, multi-PLD, time-encoded	SPM-CAT12	yes
Quantiphyse (ASL) (24)	yes	yes, using dcm2niix	NIfTI supported, ASL-BIDS in development	PASL, PCASL, multi-PLD, time-encoded, velocity-selective	FSL-FAST	yes
VANDPIRE ASL toolkit (11)	yes	yes	NIfTI not supported	PCASL, multi-PLD	FSL-FAST, or Elastix	no

*ASL, arterial spin labeling; BIDS: brain imaging data structure; DICOM, digital imaging and communications in medicine; GUI, graphical user interface; JSON, javascript object notation; multi-PLD, multi-post-labeling delay; NIfTI, neuroimaging informatics technology initiative; PASL, pulsed ASL; PCASL, pseudo-continuous ASL; PVC, partial volume correction; T1w, T1-weighted. Full details can be found under (25)*.

**Learning points:**

1. Many ASL processing pipelines are available (as listed in the OSIPI 1.1 inventory)
2. Often a GUI is available, so programming skills are not needed

**Figure 3:**
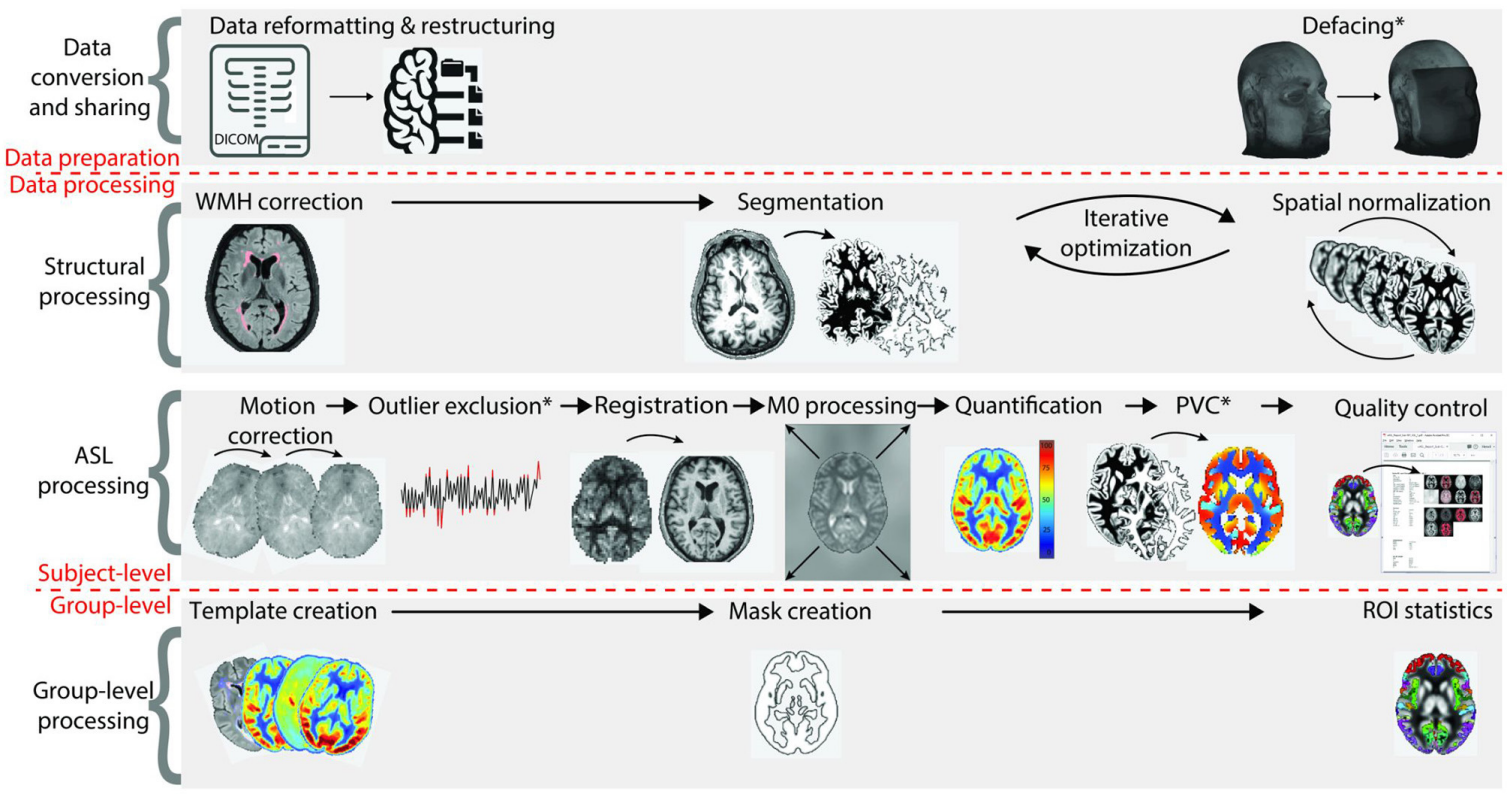
Overview of all ASL processing steps on single-subject and group-level. The first stage, data conversion and sharing, prepares data for the actual image processing. The second and third stages are structural data processing and ASL data processing (all single-subject level), respectively. The fourth stage concerns group-level processing. CBF, cerebral blood flow; PVC, partial volume correction; ROI, region of interest [adapted from (8)].

**Relevance of ASL processing steps** ASL processing can be divided into four stages, visualized in Figure 3:

1. data conversion and sharing
2. single-subject structural processing
3. single-subject ASL processing
4. group-level processing

Most steps and their order do not differ significantly between pipelines, but methodology and availability of optional steps can be variable (25).

### Data Conversion and Sharing

This step converts the MRI data as exported by the scanner into a harmonized format, organized in the defined folder structure, which facilitates standardized processing, analysis, and data sharing.

On most MRI scanners, ASL data are saved in the Digital Imaging and Communications in Medicine (DICOM) data format and are subsequently exported to research storage (PACS or “server”). However, neuroimaging analysis packages typically work with Neuroimaging Informatics Technology Initiative (NIfTI,.nii) files and are usually unable to read DICOM. Unlike DICOM, NIfTI only stores a handful of data aside from the image data, such as orientation and resolution information. Recently, the brain imaging data structure (BIDS) system was introduced to accompany the NIfTI files with an easy-to-read text file (so-called “json” file) that contains all additional scan data that are required for processing of neuroimaging MRI data (26). BIDS uses a standardized folder and file structure with an unambiguous location for multi-modal data from different subjects during different sessions. Many pipelines use NIfTI and their own system of organizing files and saving non-image data; however, using a well-defined structure - such as the one provided by BIDS and the ASL-BIDS extension - greatly facilitates subsequent processing steps.

#### Face Removal for Data Sharing

Prior to storing the converted data for further processing, many processing pipelines offer optional “defacing”, or face stripping, which removes the facial features from the 3D structural images to prevent volume reconstructions of the face, which could lead to unwanted identification of study participants. Defacing can be performed flexibly at several places in the pipeline. It is recommended to guarantee privacy before sharing data.

**Learning points:**

1. MRI data needs to be converted from DICOM to a suitable data format to allow further processing
2. Use of a standardized format (like BIDS) greatly facilitates data sharing and multi-center studies
3. Face removal in high-resolution MRI structural data can be performed to ensure participant privacy

### Structural Processing

The structural analysis part segments the brain into tissue types before registering the brain geometrically to be comparable between individuals — a process which is referred to as spatial normalization. The anatomical, usually T1-weighted images, are optionally corrected for (WM) lesions, before being segmented and spatially normalized.

#### Lesion Correction

**Figure 4:**
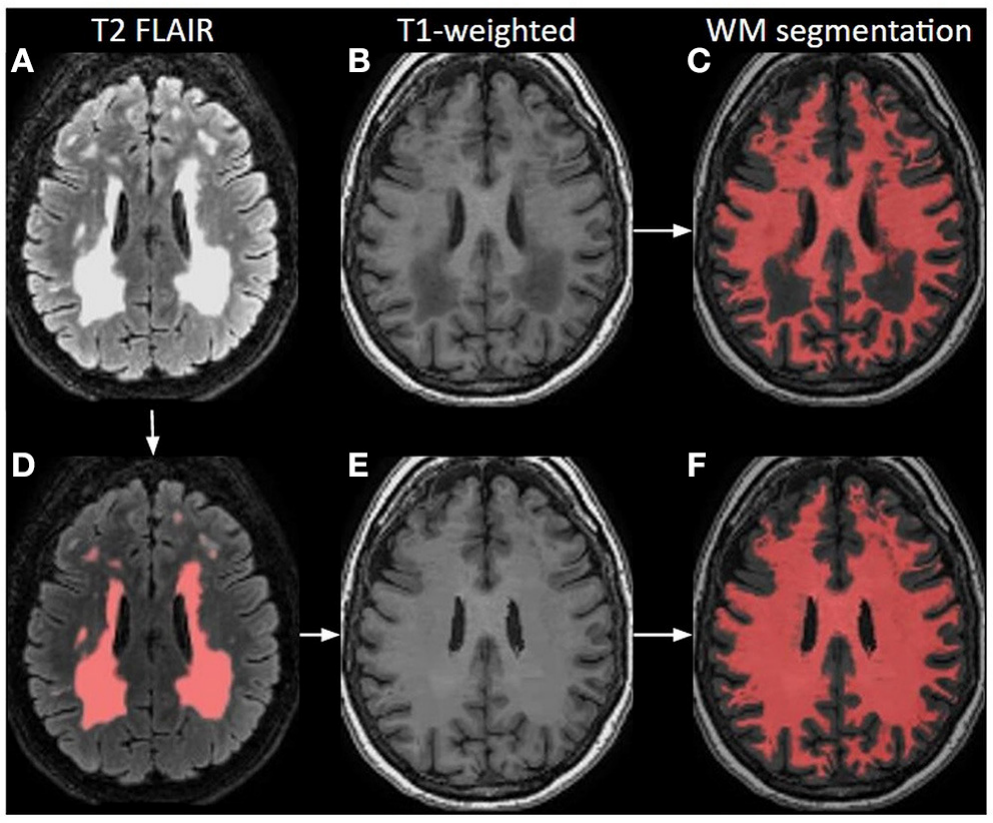
Principle of white matter lesion correction. A T2 FLAIR image **(A)** with lesion hyperintensities, corresponding to an original T1-weighted image; **(B)** and a WM segmentation image; **(C)** from a 70-year-old female. The lesions are automatically identified and segmented on the T2 FLAIR; **(D)** which are used as a mask to define hypointense regions in T1-weighted images that could be misinterpreted as GM. Based on these lesion masks, the original T1-weighted image is corrected; **(E)** and shown with the final WM segmentation overlaid in red **(F)**.

Brain lesions, specifically WM lesions, can cause erroneous T1-weighted image segmentation as WM lesions are typically hypointense comparable with GM. This can lead to incorrect brain volume measurements, registration, or even wrong estimation of GM CBF. During white-matter lesion correction, WM lesions are preferably identified based on the voxel-wise signal intensity on T2 FLAIR images, which usually have relatively poor gray-to-white matter contrast but excellent contrast between the normal-appearing WM and WM hyperintensities (Figure 4). These lesions are then used as localization reference for the corresponding hypointensities in the T1-weighted image, which are then corrected for in a process named “lesion filling”. “Lesion filling” fills the hypointense lesions on the T1-weighted image with the intensity of the surrounding normal-appearing WM.

#### Tissue Segmentation and Normalization

Segmentation and normalization are iterative, intertwined steps, as each step improves the quality of the other. They are needed to extract ROI-based CBF values from an atlas.

During segmentation, the T1-weighted structural images are divided into different segments – typically GM, WM, and cerebrospinal fluid (CSF) – by comparing the voxel-wise image intensity with the voxel-wise image intensity of a pre-segmented template brain. The most often used template brains are a variant of the template brain created by the Montreal Neurological Institute (MNI) (27). This is performed with methods such as SPM12 and FSL-FIRST (28–30) which threshold the T1-weighted signal intensity at each voxel and at the same time compare the individual brain image with the pre-segmented brain template.

Brain tissue types must be detected to allow accurate spatial normalization and registration of the single images, which forms the basis for succeeding steps such as the mean group CBF calculation or the application of standardized atlases, allowing between-subject comparisons. Poor contrast between GM and WM can cause faulty segmentation, initiating a domino effect of processing errors, resulting in wrongly estimated CBF values. Segmented images are stored and serve as anatomical references for later processing of CBF images (31).

Every brain has a different shape and size, introducing structural heterogeneity and reducing comparability between individual datasets during statistical group-level analysis. This heterogeneity can be decreased by “spatially normalizing” all structural and ASL-related data into one “standard space” - while the original volumes and dimensions are in a so-called “native space”. Spatial normalization is also crucial for longitudinal within-subject analyses, in which brain structures change over time, for example in the case of brain atrophy.

**Figure 5:**
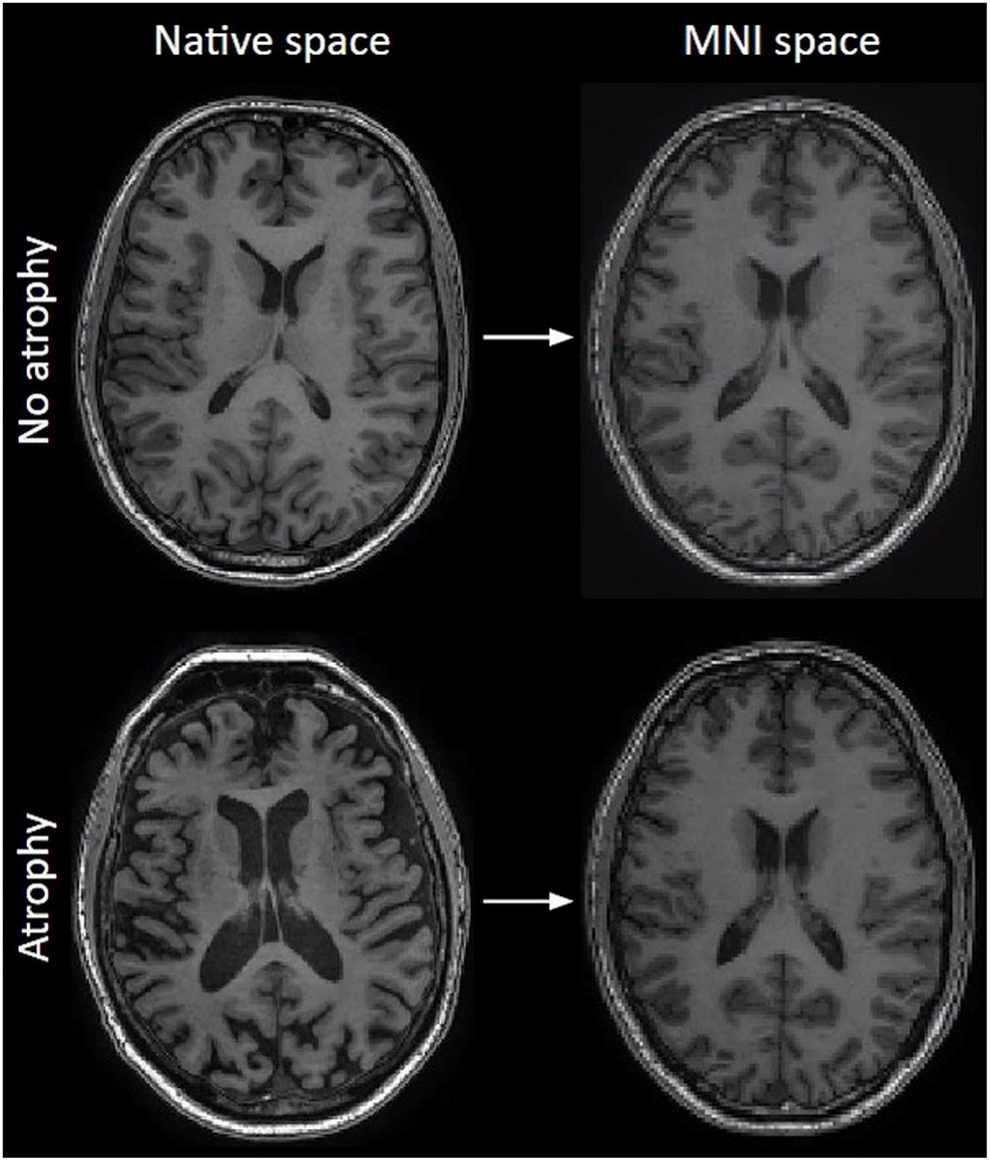
The effect of normalization on T1-weighted images. Left column: original images before normalization (native space), right column: after normalization to MNI space. Note how size differences and atrophy are largely compensated.

During spatial normalization, each part of a structural brain image is molded, kneaded, and fitted (referred to as “warped”) to the size and shape of an average brain with standard dimensions and voxel sizes, also called a template (32) (Figure 5). Several brain templates exist for different groups. The most commonly used is the MNI template, explaining the often-used expression “warp to MNI space” (33).

Spatial normalization can become difficult once the brain deviates from the usual anatomy, for example in the presence of tumor tissue, for which the use of subject-specific masks is advised. Similar to segmentation, normalization of the pediatric brain is challenging due to the immaturity of the sulci and gyri, requiring specific brain atlases. While adult-brain templates offer good performance when used on the brains of children of five years or older, and a reasonable performance even close to three years, a dedicated template is required for children of two years and below, above all neonates and fetuses (34–38). This is partly caused by incomplete myelination which, in the absence of — or even inverted — gray-to-white matter contrast, precludes segmentation (Supplementary Material 2). Currently, most pipelines only offer adult templates without a ready solution for processing ASL images of (very) young children.

**Learning points:**

1. Hypointense lesions on T1-weighted images can be filled to avoid being misclassified as GM tissue
2. Segmentation and normalization are iterative processes to separate CSF, GM and WM borders and bring all brains to the same dimensions - or standard space - for spatial comparability
3. These processes are based on normal brains and can fail in case of abnormal brains, e.g., with pathology or incomplete myelination

### Single-Subject ASL Processing

Before CBF can be quantified, the ASL image data must undergo several preparatory steps, including motion correction, outlier exclusion, registration, and M0 processing. These steps are followed by CBF quantification and optionally partial volume correction.

#### Motion Correction

**Figure 6:**
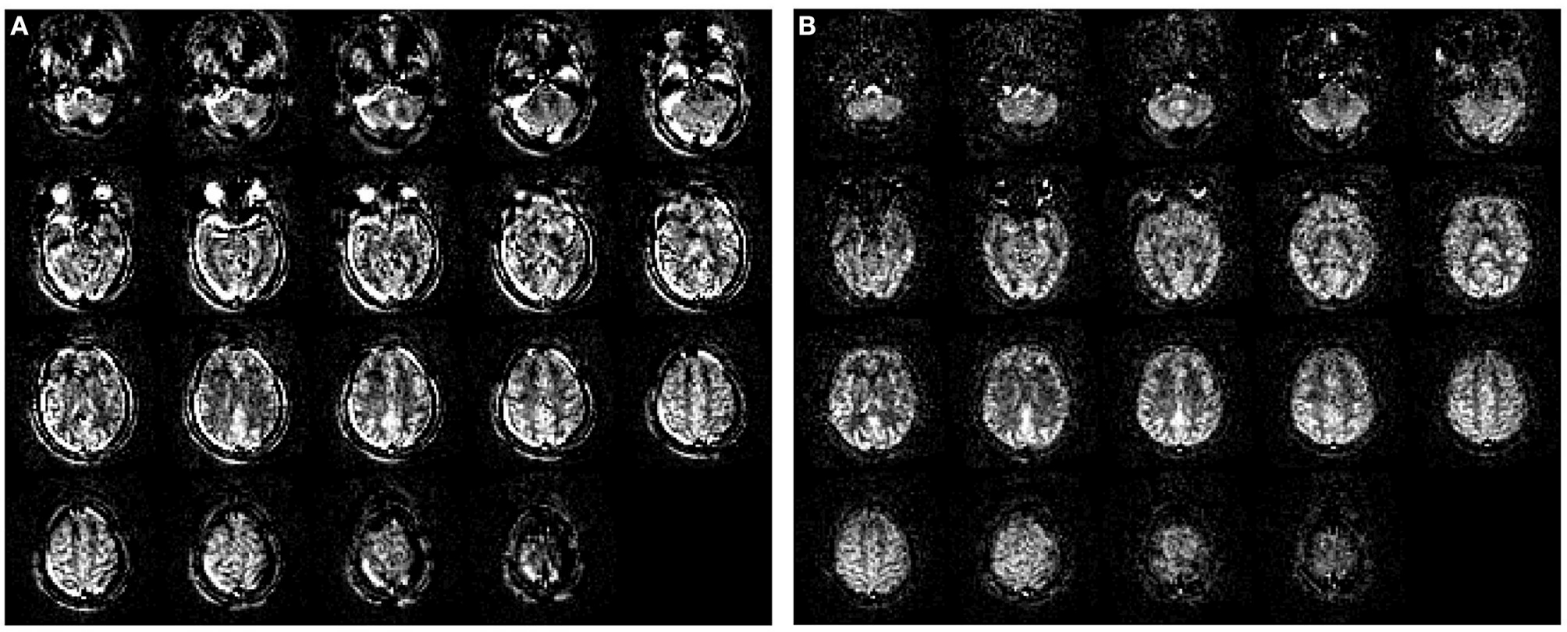
The effect of motion correction. **(A)** Perfusion image without motion correction; **(B)** The same perfusion image after motion correction. Data were obtained with pseudo-continuous labeling and 2D EPI readout without background suppression on a Siemens scanner. The original data had a single yaw rotation (subject turning the head from left to right). Motion artifacts can be easily recognized by prominent patterns of very high and very low (i.e., negative) signals next to each other in areas where the static tissue has high contrast differences — visible as rims around the brain edge and skull.

The multiple repetitions of control-label pairs first need to be aligned to each other to compensate for head motion during image acquisition. ASL is a subtraction technique where a control and label image difference is proportional to tissue perfusion. Hence, even subtle head motion can ruin the subtraction image because of the large difference in signal intensities between the brain, skull, and air, or between the GM and WM on raw (non-subtracted) ASL images. This leads to subtraction artifacts which are mostly visible as an extra rim around the brain, because of the large contrast between the head and air. Motion correction between all the control and label images is thus mandatory but can be difficult when the acquisition only has a few control-label repetitions or only outputs the average of the control-label pairs. Figure 6 depicts the effect of motion correction on image quality. Note that motion correction requires sufficient contrast in the non-subtracted ASL raw data for the algorithm to work.

#### Outlier Exclusion

Individual control-label pairs with excessive artifacts (excessive patient motion, labeling failure, etc.) can be excluded as outliers by voxel- or volume-wise comparison of individual images with the mean of all pairs when many control-label pairs exist. Because an included single control-label pair with a strong artifact can ruin the CBF calculation, excluding a relatively small number of pairs with outliers can improve the quality of the resulting CBF image. Note that because of the fewer control-label pairs available in 3D acquisitions, this is mostly done for 2D EPI acquisitions.

#### Registration of ASL to Structural Images

Motion-corrected control-label pairs — and if present, the M0 image(s) — need to be registered to the (native space) T1-weighted structural images to correct for differences in their position or angulation. This step, later on, allows both the registration of the segmented GM and WM maps to ASL images and the spatial normalization of the CBF data to the standard space, when applying the spatial normalization that had already been computed for the structural images during the structural processing stage.

Registration needs comparable image contrast in the ASL and T1-weighted images, otherwise, registration will fail or be less accurate. On the T1-weighted image, image contrast is largest between GM, WM, and CSF tissue types, and between the brain and outside the brain. Raw ASL images — or the M0 image — are low-resolution, in fact, T2-weighted images. While the intensities differ from the T1-weighted images, similar structures are visible as in the T1-weighted images. As long as the image contrast is in the same place, depicting similar tissue boundaries, newer registration algorithms can register images with different image weightings — e.g., T1- and T2-weighted. However, if a raw ASL image becomes too blurry — as can happen in a 3D spiral ASL sequence — the image contrast may be too small in the ASL image and registration may fail. In such cases, the perfusion-weighted control-label subtracted image can still be registered to the GM segmentation from the T1-weighted image. GM perfusion is around three times as high as WM perfusion, and perfusion in CSF and outside the brain is expected to be zero. Therefore, the contrast in the perfusion-weighted image is similar to the segmented GM image.

#### M0 Image Processing

With ASL we can measure absolute CBF values. But MRI images are influenced by multiple factors and generate data in arbitrary units (a.u.). To translate those a.u. to mL/100g/min, a reference image is needed for calibration. The difference image, also called delta-M or ΔM, is obtained through control-label subtraction and it is proportional to CBF but in a.u. To properly scale the delta-M image, we need a calibration index, reflecting signal, in the same a.u., from a voxel completely filled with blood water protons. This index represents the equilibrium magnetization (M0) of blood.

Because a voxel filled fully with blood is difficult to find with the relatively low ASL resolution — usually around 4x4x4 mm3 — a brain tissue M0 image is used instead. The added benefit of using an M0 image for calibration is the correction of the B1-field inhomogeneity. B1-field inhomogeneity is a smooth intensity bias across MR images, resulting from the RF-coil imperfections. Note that an M0 image is essentially a control image without background suppression.

Some ASL sequences do not contain an M0 image. A reasonable assumption is that the quantification (i.e., the scaling of delta-M in a.u. to CBF in mL/100g/min, see below) is equal across the brain; therefore delta-M can be used as a relative CBF estimate. Normalization of delta-M to whole-brain GM or WM CBF, or relative to the contralateral hemisphere, is then recommended.

#### CBF Image Quantification

The actual perfusion-weighted image is calculated by subtracting the label from the control images. This subtraction image is “quantified” — i.e., converted to physiological values — to obtain a CBF image in mL/100g/min (4) using the M0 image described above. The most commonly used model for CBF quantification (4) assumes that all the labeled blood has arrived in the imaging voxel before the start of the readout (i.e., the arterial transit time (ATT) from the labeling plane to the readout slice is lower than PLD), and has stayed intravascularly and decayed with the blood T1 — the halftime of magnetization relaxation specific to blood, e.g., ~ 1.65 seconds at 3 tesla. Unfortunately, this single-PLD model is sensitive to the ATT of the labeled blood, implying the need for a sufficiently long PLD. If the PLD is shorter than the ATT, macrovascular artifacts (i.e., labeled blood in proximal arteries rather than the distal capillaries or tissue) and so-called signal void (i.e., the labeled blood has not arrived yet in distal voxels leaving an empty area on the map) will appear. This issue can be addressed, in the majority of cases, by using a multi-PLD or time-encoded ASL acquisition (39). With multi-PLD sequences, ATT can be measured to improve the accuracy of CBF estimation (40). These sequences are now slowly becoming available for clinical use and some of the pipelines are already supporting their quantification.

Accurate CBF image calculation requires the successful completion of all previous calculations and registration steps. Several factors, such as labeling duration and the blood T1, can influence the CBF quantification. Therefore, these must be correctly taken into account by the quantification part of the image processing pipeline. Incorrectly defined values of these parameters strongly affect the CBF results, as demonstrated in Table 2. For example, the incorrectly entered lower PLD will cause underestimation of the magnetization decay and subsequently of CBF (row 2 of Table 2). Entering a shorter labeling duration than the true value (row 3 of Table 2) will lead to an overestimation of CBF. Also, low hematocrit, influencing the blood T1 relaxation, will lead to an overestimation of CBF, if normal hematocrit values are assumed by the model, as illustrated for blood T1 itself in Table 1 (row 4 of Table 2).

**Table 2:** Model calculation of quantification input parameters effect on CBF calculation.

**Parameters**	**mean GM CBF (mL/100 g/min)**	**mean WM CBF (mL/100 g/min)**
Real parameters: PLD: 2,025 ms, LD: 1,450 ms, blood T1: 1,650 ms, PVC	53.3	21.7
Falsely reduced PLD: **PLD: 1,800 ms**, LD: 1,450 ms, blood T1: 1,650 ms, PVC	46.5	18.9
Falsely reduced LD: PLD: 2,025 ms, **LD: 1,200 ms**, blood T1: 1,650 ms, PVC	60.3	24.5
Falsely reduced blood T1: PLD: 2,025 ms, LD: 1,450 ms, **blood T1: 1,450 ms**, PVC	66.4	27.0
Without PVC: PLD: 2,025 ms, LD: 1,450 ms, blood T1: 1,650 ms, **no PVC**	40.3	21.3

*Starting from the correct quantification, GUI parameters input in row 1, PLD, LD, blood T1, and omission of PVC were subsequently used incorrectly to demonstrate their effects of each modulation. PLD, post-labeling delay; LD, labeling duration; blood T1, T1 relaxation of blood; PVC, partial volume correction*.

The model introduced above, called “one-compartment”, assumes that a voxel only has a single intravascular “compartment” composed fully of blood. An extended model can either assume an instantaneous exchange of blood label to tissue upon arrival and accounts for a faster T1 relaxation of the label within the tissue (~1.2 s for GM and ~ 0.9 s for WM at 3T) than within the vessels. A fully “two compartmental” model also accounts for signal from extravascular water in tissue within the same voxel and models signal behavior in both compartments and a transfer between them. New sequences using a dedicated diffusion-weighted or multi-echo time ASL can then measure the transport across the blood-brain-barrier using such two-compartment models (41–43).

#### Partial Volume Correction

The CBF value in each voxel is a mix of GM and WM CBF, and potentially even CSF CBF (which should be zero), as each voxel's volume is partially GM, WM, and CSF. Because the CBF in GM is around 2 to 4 times higher than the WM CBF, this so-called tissue partial volume significantly affects the CBF value in each voxel. As these partial volume effects (PVE) can differ between groups that shall be compared, it is a source of bias if not corrected. While each MRI sequence suffers from partial volume effects to some extent, ASL's relatively low spatial resolution — typically around 4x4x4 mm3 full-width-half-maximum (FWHM) — makes this effect around 64 times as strong as a typical 1x1x1 mm3 FWHM 3D T1-weighted structural image. This is why GM, WM, and CSF segmentations from the structural images are typically used to correct these PVE in ASL images.

The simplest option to account for partial volume effects is to define a ROI in which the average GM CBF is calculated, based only on voxels that have a high partial volume of GM (e.g., more than 70%) (44). However, in cases of too thin cortical GM in patients or elderly with atrophic brains, this will leave very few voxels that can be included when calculating GM CBF (45). This significantly decreases the SNR and consequently the statistical power of an analysis. Lowering the GM threshold would widen the ROI, but it would decrease measured GM CBF, due to the higher presence of WM CBF in the GM ROI. A more robust way to correct for PVE is to use partial volume correction, a technique where the GM CBF is estimated in each voxel by regressing out the GM and WM volumes from the CBF image (Table 2) (46). Note that in cases of incorrect segmentation or registration, all forms of dealing with PVE are equally affected (44).

#### Quality Control

Many processing software packages provide quantitative quality control (QC) parameters, allowing the user to evaluate the individual and group-level quality of the data itself, and of the individual image processing steps. In case this information is not provided by the pipeline, it is advisable to visually evaluate the data in order to detect possible acquisition and processing errors that might impact further analysis. Potential quantitative QC parameters include estimations of head motion or misalignment (both in mm) or SNR.

**Learning points**

1. Motion can severely degrade the quality of ASL CBF images, but can often be corrected using motion correction techniques
2. Corrupted control-label pairs may be excluded from creating the average CBF image using an outlier exclusion step
3. Image registration of ASL to structural images is important for deriving CBF to anatomical regions, but it demands a similar contrast of each image
4. The M0 image, or a control image without background suppression, allow the scaling of the perfusion-weighted images to receive CBF in mL/min rather than a.u.
5. The perfusion-weighted CBF image is also called the delta-M image
6. Partial volume effects can decrease GM CBF values. Partial volume correction can alleviate this effect, especially in the case of cortical atrophy.
7. Quality control of individual or group-level results is highly recommended to ensure validity

### Group-Level Processing

A few image processing steps can be performed on a group level, such as the creation of a template and a group-analysis mask.

#### Template Creation

Group templates are group-average images of CBF that can be useful to inspect any consistent differences between subgroups (e.g., per MRI scanner, per time point, etc). The template creation produces one group- or group-average image. This can be an average CBF image, but can also be an average ATT image, mean control image, M0 image, or T1-weighted image. Additionally, sub-group templates can be created, for example, CBF images for each sex, or for pre- and post-treatment in interventional studies.

#### Mask Creation

To optimize the statistical analysis on group-level, the creation of a dedicated group analysis mask is recommended. An analysis mask defines which voxels will be included or excluded in the analysis. By default, the analysis mask includes both GM and WM. Excluded regions are: (a) outside the brain or field-of-view; (b) instrumental artifactual values such as the signal drop in a susceptibility area; and (c) physiological artifactual values due to vascular fluctuation. As the presence of such unwanted properties may vary between individuals, the group analysis mask is adjusted to include only voxels that are part of the individual analysis mask of at least 95% of the individuals in the study group. The masks are mainly based on GM regions from structural images but shall exclude outliers on CBF images.

#### ROI Statistics

CBF values for the whole brain, GM, and predefined regions of interest are calculated for each individual as well as for the entire group, in the ROI statistics step. Usually, the ROI can be defined by choosing one of the implemented brain atlases, such as the MNI atlas (which is not the template), dividing the brain into standardized, specific sites within the brain. The resulting CBF values are stored in a data file, ready for further statistical analysis.

Calculation of CBF values in specific regions is optional depending on the study goals, e.g., to compare the CBF in the left parietal cortex of dementia patients vs. healthy controls. There are multiple atlases providing different ROIs for different groups which can be used to extract CBF values from specific brain regions, but which should be chosen as a match to the examined group (47). It is important to understand that many brain structures are either too small to be analyzed due to the limited spatial resolution of the ASL sequence (e.g., brainstem nuclei), or are situated in areas affected by susceptibility artifacts (mainly located near tissue-air transitions, e.g., the temporobasal area), or lie in an area of low SNR in the WM (e.g., the basal ganglia). Analysis masks and PVC can only account for these problems to a certain degree. Sometimes several factors apply that make an ROI-based analysis practically feasible, but the results might not be trustworthy. There is unfortunately no agreement or “rule of thumb” regarding spatial resolution and the size of ROI needed for trustworthy results.

**Learning points:**

1. Template creation delivers a group mean image, e.g., of the CBF image
2. Analysis masks define the parts of the image which shall contribute to the measurements excluding, e.g., blood vessels
3. ROI statistics are atlas-based and deliver CBF values and other statistical information of anatomical structures

## The ASL Processing Outcome

Depending on the ASL analysis software, different approaches to both visualize or report ASL-related outcome parameters can be implemented.

The perfusion-weighted images, created after the control-label subtraction, but before quantification using the M0 image calibration, provide non-measurable, unitless images, only allowing visual, qualitative evaluation. More valuable are the calibrated, quantitative CBF images. In a study, one will usually receive CBF information on a single-subject as well as on a group level, comparing two or more groups. These CBF images should be used for further statistical ROI- or voxel-wise group-based analysis. Calibrated CBF images are typically presented in a grayscale version. Additional image files might be produced, such as images of the segmented GM and WM structures, arterial transit time (ATT, in seconds) in case of multi-PLD ASL, or the spatial coefficient of variation (sCoV, in %), a relative measure of signal heterogeneity (48).

Besides visual output, many ASL-processing software packages provide data files with quantitative values containing the measurement results of the ROIs, but also other parameters such as brain volume in mL. Finally, some software packages allow basic mathematical calculations or even the creation of graphs.

## Conclusion

Clinical ASL processing applications are continuously expanding. In this educational review, we provide an overview of the technique ASL and all the necessary steps to run a successful data analysis for research purposes without prior knowledge of ASL-adapted image processing, to help researchers start their own ASL-based studies.

## Author Contributions

The contribution of the co-authors is distributed as follows: PC, JP, MP, AJ, FB, HM, and VK: article concept. PC, JP, MD, BP, MP, LJ, NP, FB, HM, and VK: writing of the first draft. PC, JP, MD, BP, SD, HM, and VK: delivery of image content. PC, JP, MD, BP, MP, SD, LJ, NP, LH-G, AJ, JK, FB, HM, and VK: critical evaluation of the draft and approval of the submitted draft. The responsibility for the entire manuscript is shared between HM and VK. All authors contributed to the article and approved the submitted version.

## Funding

This project is part of the Eurostars-2 joint programme with co-funding from the European Union Horizon 2020 research and innovation programme (ASPIRE E!113701), provided by the Netherlands Enterprise Agency (RvO). MD, JP, and HM are supported by the Dutch Heart Foundation (03-004-2020-T049). BP, JP, and HM are supported by the EU Joint Program for Neurodegenerative Disease Research, provided by the Netherlands Organisation for Health Research and Development and Alzheimer's Netherland (DEBBIE JPND2020-568-106). FB is supported by the NIHR biomedical research center at UCLH. SD is supported by grants from the National Institutes of Health (R01 NS111115 and R03 AG063213). NP is funded by the Dent family foundation. PC, JP, MD, BP, VK, and HM are members of Task Forces of COST Action CA18206 Glioma MR Imaging 2.0, supported by COST (European Cooperation in Science and Technology) www.cost.eu; www.glimr.eu.

## Conflict of Interest

The authors declare that the research was conducted in the absence of any commercial or financial relationships that could be construed as a potential conflict of interest.

## Publisher's Note

All claims expressed in this article are solely those of the authors and do not necessarily represent those of their affiliated organizations, or those of the publisher, the editors and the reviewers. Any product that may be evaluated in this article, or claim that may be made by its manufacturer, is not guaranteed or endorsed by the publisher.

## Supplementary Material

Supplementary Material 1 ASL abbreviation directory.

Supplementary Material 2 Spatial normalization in a 12 month-old and a 5 year-old, respectively using different dedicated templates of different sizes as superimposed in the center (red: GM segment in the 5-year-old, green: GM segment in the 1-year-old). Note the comparably poor GM-WM contrast in the one-year-old. Due to incomplete myelination and therefore poor GM-WM contrast the segmentation in children especially below 1 year remains a challenge with standard sequences.

## Abbreviations

ASL: arterial spin labeling
BIDS: brain imaging data structure
CBF: cerebral blood flow
DSC: dynamic susceptibility contrast
DICOM: Digital Imaging and Communications in Medicine
FLAIR: fluid-attenuated inversion recovery
GM: gray matter
GUI: graphical user interface
JSON: JavaScript Object Notation
M0: magnetization (of blood) at equilibrium, a.k.a. the calibration image
MRI: magnetic resonance imaging
multi-PLD: multi-post-labeling delay
NIfTI: Neuroimaging Informatics Technology Initiative
PASL: pulsed ASL
PCASL: pseudo-continuous ASL
PLD: post-labeling delay
PVC: partial volume correction
PVE: partial volume effect
ROI: region-of-interest
SNR: signal-to-noise ratio
WM: white matter.
