# Supplementary material for: A Beginner's Guide to Arterial Spin Labeling (ASL) Image Processing
Source: Front Radiol. 2022 Jun 14;2:929533. doi: 10.3389/fradi.2022.929533 (PMC10365107; doi:10.3389/fradi.2022.929533)
Supplement: Supplementary Material 1 — ASL abbreviation directory. [file Data_Sheet_1.PDF]

Online supplement 1: ASL terminology dictionary

This brief dictionary was created to facilitate the reading of the educational review. More definitions can be found in the ASL Lexicon prepared by OSIPI Task Force 4.1 (https://osipi.org/task-force-4-1/).

ATT: The arterial transit time (**ATT**) describes the time in (milli)seconds for blood to travel from the location of the labeling plane in the brain feeding arteries to the image level in the brain. **ATT** should not be confused with **PLD**, which is an MRI sequence parameter set by the user in agreement with an assumed **ATT** value.

Atlas (also see **template** for comparison)**:** An **atlas** is formed by the brains of a group of humans and defines the average locations of brain structures and their boundaries, or average values of parameters like perfusion.

BIDS: Brain Imaging Data Structure (**BIDS**) is a standard for organizing imaging data to facilitate data sharing and post-processing (https://bids.neuroimaging.io/index.html). An extension to ASL, ASL-BIDS, was included in **BIDS** 1.5.0, and contains the most important ASL metadata (https://psyarxiv.com/e87y3).

Compartments: We assume a number of **compartments** to be within a voxel, in which we will model the label signal decay, e.g., a tissue **compartment** and a vascular **compartment**. The number of **compartments** is one in most pipelines, assuming that all labeled blood resides within arteries and arterioles.

DICOM: Digital Imaging and Communications in Medicine (**DICOM**; file extension .dcm) is the globally used data format to save radiological images. It is the most common format to export data from the MRI scanner to a central server or portable device. **DICOM** format can contain a lot of metadata, but metadata such as acquisition parameters can be missing for advanced sequences such as ASL.

JSON: JavaScript Object Notation (**JSON**) is a text-based format to save structured metadata in a format that is relatively efficiently structured while still human-readable. In the ASL-BIDS format, each **NIfTI** image file is accompanied by a so-called **JSON** “sidecar” file containing ASL metadata.

Labeling duration: Labeling duration defines the (temporal) duration of the labeling. Longer **labeling duration** results in a larger blood label ‘bolus’ and a higher signal-to-noise ratio. It defines the duration during which labeling pulses are applied in PCASL or the time between labeling and label saturation (i.e., the label is nulled after a certain time period) in PASL.

Labeling distance: The **labeling distance** is the distance in millimeters between the center of the imaging volume and the center of the labeling plane (this term is typically used for PCASL). An alternative term is the **labeling gap**, which is the nominal gap between the leading edge of the labeling slab and the closest edge of the imaging volume (this term is typically used for PASL). The placement of the labeling plane can affect the **labeling efficiency** (e.g. placement on tortuous vessels, or at an angle differing significantly from perpendicular to the vessel length can reduce the **labeling efficiency**).

Labeling efficiency: Labeling efficiency is defined as the efficiency of the labeling pulse to invert the blood magnetization. A default value of 0.85 is assumed for PCASL and 0.98 for PASL. Furthermore, the loss of label caused by background suppression pulses is often included in the **labeling efficiency** by multiplying the ratio by ~0.93 for each background suppression pulse (e.g. the total **labeling efficiency** for a PCASL with 2 background suppression pulses is 0.735 = 0.85 * 0.93 * 0.93).

M0 image: The **M0 image** is an independent scan within the ASL sequence to determine the basic tissue magnetization of the scanned brain volume. Besides the absence of labeling and background suppression pulses, and possibly different TR, an M0-scan should be acquired with the same parameters and sequence as the ASL images. It is used for CBF quantification.

Mask: A **mask** is a filter applied to the entire image defining which voxels will be included or excluded in the analysis. Usually, **masks** are used to cut out unwanted elements of data such as vessels or pixels with signal values outside a certain range of expected values.

NIfTI: The Neuroimaging Informatics Technology Initiative (**NIfTI**, https://nifti.nimh.nih.gov) file format is a data format (file extension .nii or nii.gz for the compressed version) that can efficiently contain multi-dimensional image data. It is the standard format used by most research image post-processing programs such as SPM or FSL. This format, however, does not allow storing further personal or sequence metadata.

PLD: The post-labeling delay (**PLD**) is an acquisition setting for the time between the labeling of the blood in the labeling plane and the image acquisition of the brain. The **PLD** should be a compromise between too short delay (leading to incomplete label arrival and macrovascular artifacts) and too long delay (leading to loss of signal due to T1w label relaxation). Typical values are around two seconds, but this depends on the subject's age and pathology.

PVC: Partial volume correction (**PVC**) deals with the problem that image voxels in ASL are typically large enough to contain multiple tissue types. These tissue types have distinctly different CBF. **PVC** methods use a map of tissue volume percentages, frequently obtained from segmenting high-resolution T1-weighted images, to resolve the issue with tissue mixing at the cost of reducing the ASL resolution.

RF pulse: A radiofrequency (**RF**) electromagnetic pulse (in megahertz, MHz) is the classic technique in MRI to change the magnetic status of tissue under examination, which will eventually lead to the MRI image. It is generated by the MRI scanner.

ROI: A region of interest (**ROI**) is a predefined, subject- or group-specific, region within an image. Examples include a lesional **ROI** (a part of a brain tumor), a functional **ROI** (the motor cortex), anatomical **ROI** (the frontal lobe), or a vascular **ROI** (the posterior vascular territory). An **ROI** analysis is used when

an **ROI** is hypothesized to be affected, contrasting with a non-specific whole-brain voxel-based analysis (VBA).

Slice readout time: The **Slice read-out time** of a 2D multi-slice acquisition scheme — also called the inter-slice time — is the time between the start of the acquisition of successive slices. This information is essential in CBF quantification to compensate for the acquisition time of sequential slices, and thus for obtaining the effective **PLD** of each slice, as brain slices are acquired sequentially at different times.

SNR: The signal-to-noise ratio (**SNR**) defines the quality of the MR signal. The “noise” represents all unwanted sources outside the target object under examination, while the “signal” represents the desired sources from, e.g. the examined body part’s tissue. High **SNR** is desired to have a reliable measurement performance, while a low **SNR** means that images are of poor quality.

Template: The term **template** describes a geometric coordinate system (or standard space) as a stereotactic frame for MRI data. It is based on measurements in many different brains. A template is used as a reference for brain spatial normalization defining *where* structures are located. This differs from the atlas, which specifies the anatomical relation of predefined regions of interest (**ROI**s) based on a group of individual brains. Example: The Montreal Neurological Institute template (**MNI template**) is a stereotactic space. Individual MRI scans are enlarged or shrunk (“warped”) to make individual structures fit into the dimensions of this common standard space. When warped to the standard space, as opposed to the original native space, individual images become comparable. This is called spatial normalization.
