# Supplementary figures and images for: A Beginner's Guide to Arterial Spin Labeling (ASL) Image Processing
Source: Front Radiol. 2022 Jun 14;2:929533. doi: 10.3389/fradi.2022.929533 (PMC10365107; doi:10.3389/fradi.2022.929533)

Native space

MNI space

12-month-old

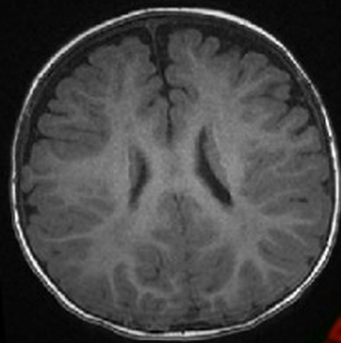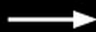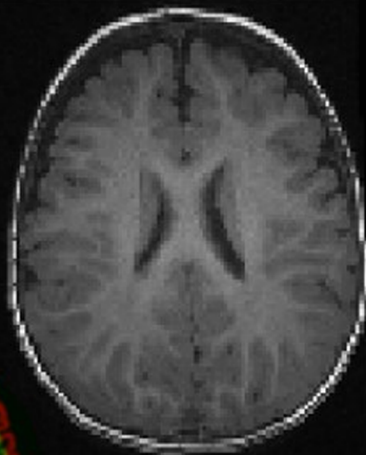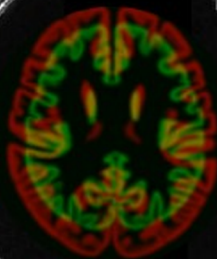

5-year-old

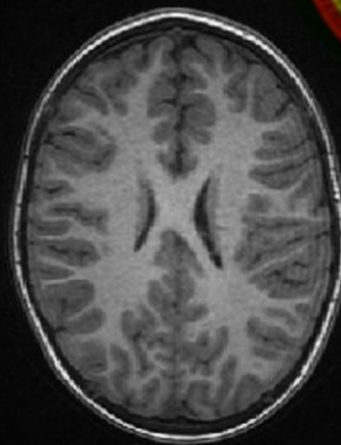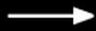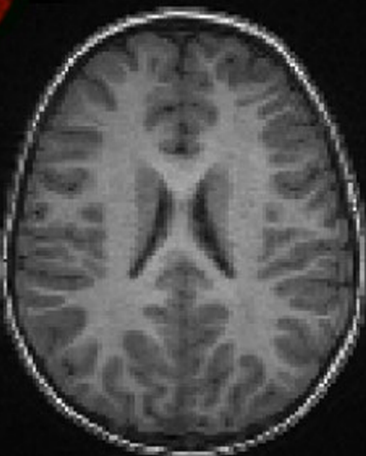

Supplement: Supplementary Material 2 — Spatial normalization in a 12 month-old and a 5 year-old, respectively using different dedicated templates of different sizes as superimposed in the center (red: GM segment in the 5-year-old, green: GM segment in the 1-year-old). Note the comparably poor GM-WM contrast in the one-year-old. Due to incomplete myelination and therefore poor GM-WM contrast the segmentation in children especially below 1 year remains a challenge with standard sequences. [file Data_Sheet_2.PDF]
